# Short lifespan under dietary cholesterol depletion is associated with gut dysfunction in *Drosophila melanogaster* females

**DOI:** 10.1038/s41514-026-00341-5

**Published:** 2026-02-19

**Authors:** Brooke Zanco, Christen K. Mirth, Carla M. Sgrò, Matthew D. W. Piper

**Affiliations:** 1 School of Biological Sciences, Monash University, Melbourne, VIC Australia; 2 Division of Biosciences, University College London, London, UK

**Keywords:** Physiology, Zoology

## Abstract

Dietary restriction may extend lifespan by improving late-life gut health. Because micronutrients mediate the effects of macronutrient ratios on longevity, we examined how cholesterol limitation affects gut health in female *Drosophila melanogaster*. Low-cholesterol diets increased intestinal permeability and reduced lifespan, however, not all flies lost barrier function before dying. This indicates gut dysfunction is either a marker of ageing, or contributes to death, but predominantly during dietary cholesterol limitation.

## Sterol-dependent gut integrity as a candidate determinant of female *Drosophila* longevity

Deciphering how the deterioration of specific organs determines lifespan remains a critical challenge in ageing research^1–3^. One organ thought to be central in mediating lifespan is the gut^4^. This is thought to act via epithelial barrier dysfunction, which results in a leaky gut phenotype^4^. These effects have been predominantly reserved to females in *Drosophila*^5^, and are thought to be driven by female specific gut remodelling in response to mating and diet^6–8^.

Dietary restriction (DR), whereby food intake is reduced without malnutrition, has been shown to extend lifespan across a broad range of taxa^9–13^. Given age-associated gut pathologies are responsive to diet^5,14,15^, it is possible that DR may extend lifespan by reducing gut pathology and in doing so, preserve late life gut health.

Our recent work has established that an essential dietary micronutrient, a sterol, is a key determinant of lifespan in response to DR in *Drosophila* females^16^. These data showed that lifespan is longer on lower protein (or DR) diets predominantly because the flies have lower reproduction, which may rescue them from overcommitting sterols into higher rates of egg production^16–19^. Thus, if gut integrity is key to longer life, the use and allocation of dietary sterols between somatic tissue and eggs may mediate the occurrence of later life gut pathologies in *Drosophila* females.

## Gut integrity declines more rapidly as dietary cholesterol is diluted, which is associated with a shortened lifespan

**Fig. 1:**
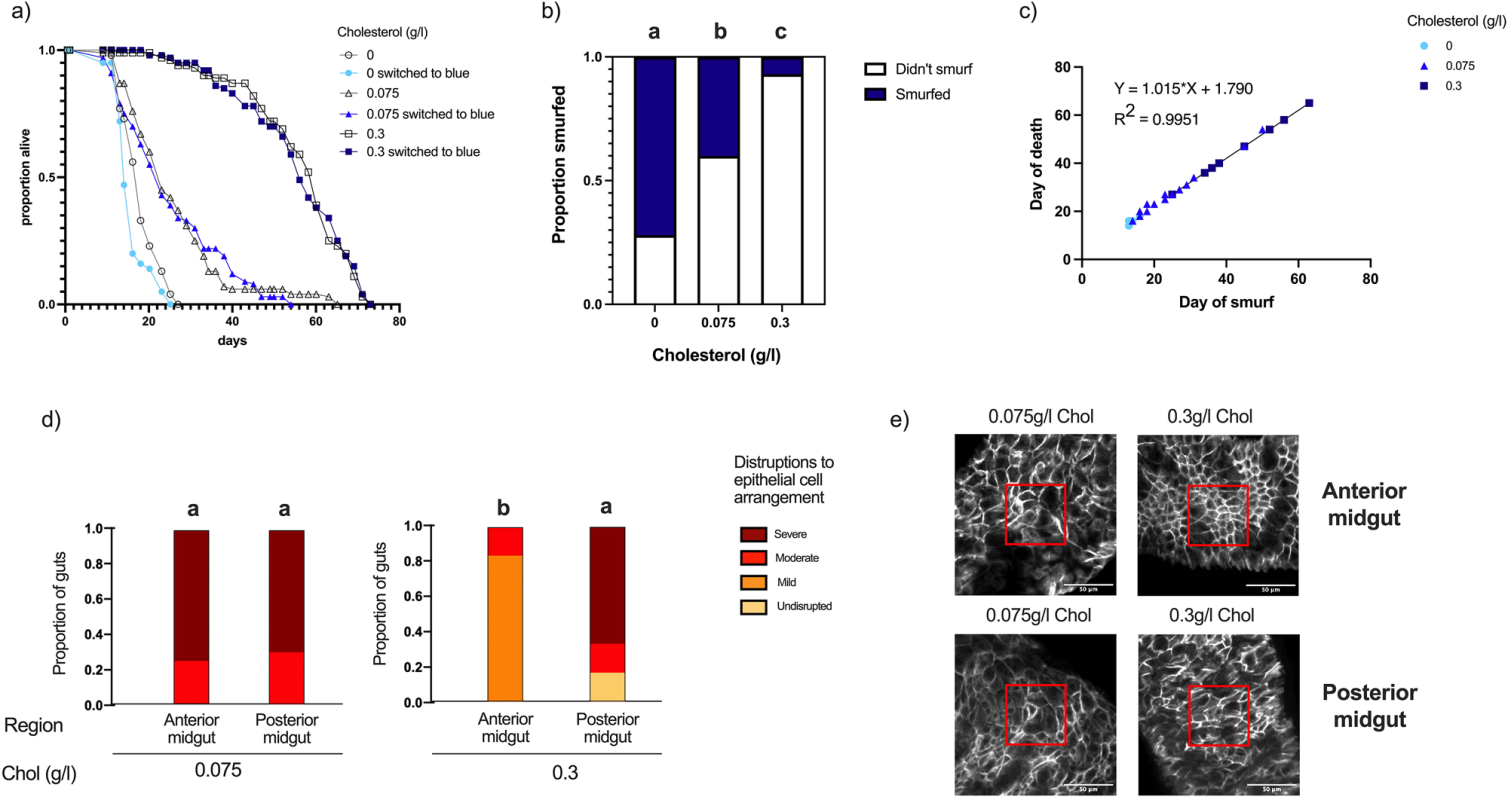
Flies fed low cholesterol diets have greater levels of gut permeability and epithelial cell disorganisation in the anterior midgut than those fed high cholesterol diets. **a** Lifespan was maximised on a high cholesterol diet (0.3 g/L) and declined as the cholesterol concentration was reduced (0.075 g/L and 0 g/L). Blue dye did not have a significant effect on lifespan (*p* = 0.060). **b** Cholesterol has a significant effect on smurfing in flies prior to death (*p* < 0.001). Letters indicate statistically distinct groups based on Tukey-adjusted pairwise comparisons (Supplementary Table 3). **c** In flies that do smurf, death occurs within ~ 48 h of turning blue, irrespective of dietary cholesterol intake. **d** Epithelial cell disruptions of the Drosophila anterior midgut at 18 days of age were significantly greater in flies fed low cholesterol diets when compared with flies that were fed an adequate supply of cholesterol. In contrast, the posterior midgut did not present with significantly different classifications of epithelial disruption across both cholesterol treatments. Letters indicate statistically distinct groups based on Tukey-adjusted pairwise comparisons (Supplementary Table 5). **e** Red boxes show the area of 150 µm × 150 µm (centred) which was selected using Fiji and then cells in this region were scored for degree of disorganisation, adapted from criteria published previously^5^. For lifespans, ten vials containing 10 mated females each were used per treatment; epithelial analyses included at least 3 females per treatment.

To test whether dietary sterol limitation affects gut integrity, we fed flies a holidic diet containing 0, 0.075, or 0.3 g/L cholesterol, all with a standard protein-to-carbohydrate ratio used in our lab^20^. We selected 0.3 g/L as the upper limit because higher concentrations do not affect egg production or lifespan^16^. From day 12, FD&C Blue Dye No.1 was added to the food to detect loss of gut barrier function, which is indicated when the dye leaks from the gut into the body cavity, turning the entire fly blue, referred to as “smurfing”^21^. As dietary cholesterol was diluted in the diet, lifespan shortened and smurfing became more frequent (Fig. 1a, b; Supplementary Tables 1–3).

We found that 72% of flies on diets with no added cholesterol (0 g/L) smurfed before death, with a median lifespan of 15 days (Fig. 1a, b). This fell to 40% smurfing and a lifespan of 24 days on diets with 0.075 g/L cholesterol, and just 7% smurfing on the highest concentration (0.3 g/L), where lifespan reached 57 days (Fig. 1a, b). The low smurfing rates at high cholesterol levels indicates a lower rate of gut permeability before death. Alternatively, lower smurfing before death could be caused by reduced feeding on higher cholesterol diets, but this is unlikely given that recent data shows no food aversion with the cholesterol levels we used^22^. Notably, when smurfing did occur, it consistently appeared ~48 h before death across all diets (Fig. 1c), suggesting a threshold effect whereby critical gut permeability marked the point of imminent death.

We examined the effects of cholesterol limitation on both the anterior (R1 and R2) and posterior regions (R4 and R5) of the midgut^23^ in flies that were 18 days old—the point at which death commences (Fig. 1d, e). The midgut was selected as we know that both the cellular structure and arrangement of epithelial cells in this region are modified by diet^5^. We found that gut permeability is likely driven by region-specific epithelial disorganisation, which we observed significantly more often in the anterior but not posterior midgut of flies fed diluted cholesterol diets compared to those fed adequate cholesterol (Fig. 1d, e; Supplementary Tables 4, 5).

Together, these results suggest that gut integrity declines more rapidly as dietary cholesterol is diluted, and this decline is associated with a shortened lifespan. While we did not assess smurfing in males, previous studies have shown that cholesterol depletion does not limit male lifespan^7^, and males do not typically smurf when fed high-protein diets^5^. Moreover, female flies that do not produce eggs, due to genetic or pharmacological interventions, live longer on high-protein, low-cholesterol diets and show reduced smurfing under sterol limiting conditions^7,24^. These findings support the idea that cholesterol investment in egg production is a major driver of cholesterol-dependent lifespan reduction^16,18^. Thus, our results likely reflect a sexually dimorphic effect of nutrition on lifespan and reproduction in *Drosophila melanogaster*^5–7^. Future work should examine the effects of specific sterol esterases, such as *Hsl* activation, on gut integrity and lifespan^17^. Additionally, temporal imaging of gut permeability or targeted sterol supplementation after intestinal barrier loss could reveal whether restoring cholesterol can rescue lifespan or if a point of no return exists.

## Intestinal barrier dysfunction is unlikely to be a universally conserved mechanism of ageing-related mortality

**Fig. 2:**
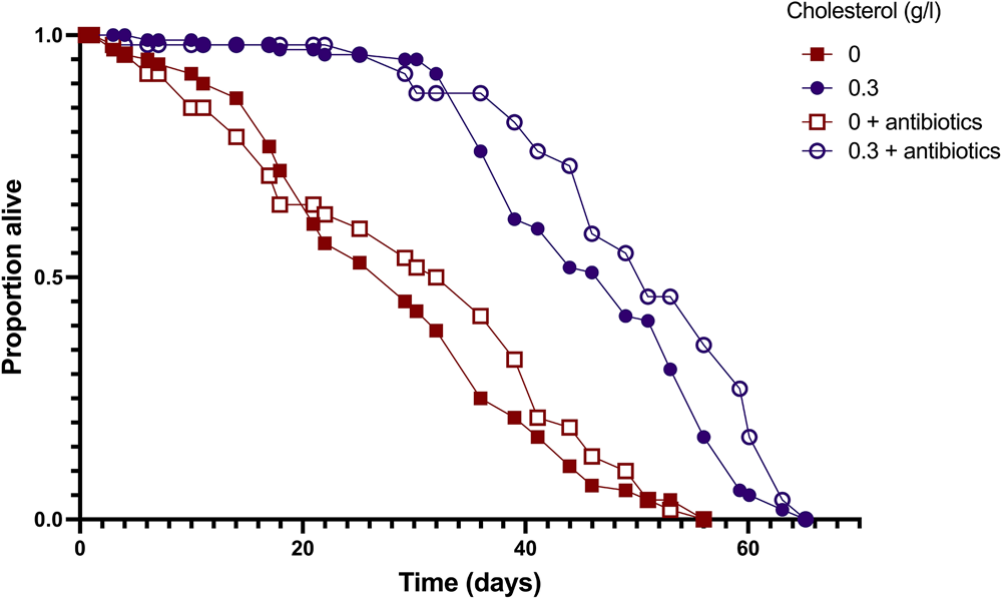
Antibiotic treatment in cholesterol-limited flies does not rescue lifespan. Flies were fed throughout life on either a cholesterol sufficient (0.3 g/L) or cholesterol limiting diet (0 g/L), with or without antibiotics. There was a significant effect of both antibiotics (*p* = 0.003) and cholesterol (*p* < 0.001) on lifespan, but no interaction (*p* = 0.872) (Supplementary Table 6). 10 vials containing 10 mated females were used in each treatment.

Reduced gut barrier function may cause death by allowing bacteria to infiltrate the body cavity, causing sepsis. Thus, we next asked if antibiotic treatment could increase the lifespan of flies fed low cholesterol diets. Treatment with five antibiotics that have been shown to clear bacteria that normally associate with *D. melanogaster* larvae^25,26^ had a small, significantly positive effect on lifespan, but did not modify the way that cholesterol restriction shortened lifespan (Fig. 2, Supplementary Table 6). This indicates that death upon cholesterol depletion is not due to illness caused by bacteria. Instead, our findings indicate that loss of gut barrier function either causes death by other means (e.g. loss of absorptive capacity) or gut permeability is a symptom, rather than a cause, of the progressive increase in mortality in these flies.

Although we observed a strong association between smurfing and time to death, this link appears specific to cholesterol depletion for most, but not all, individuals in our genetically diverse population. Intestinal barrier dysfunction has been associated with ageing across a broad range of taxa^4^, but our data indicate it may not represent a general cause of age-related death. Indeed, we found that most flies that died of old age under fully fed conditions did not smurf, consistent with previous studies showing that lifespan and gut permeability can be uncoupled^5,7,27,28^. It is also possible that for some genotypes gut dysfunction under cholesterol depletion reflects a downstream effect of systemic physiological decline. Thus, while gut permeability may signal imminent death in specific contexts, it may not be a universally conserved mechanism of ageing-related mortality.

## Methods

### Lifespan assays

All experiments were conducted using a wild type *Drosophila melanogaster* strain called Dahomey^29^. These flies are maintained in large numbers with overlapping generations to maintain genetic diversity. This means that each individual in the experiment represents a different genotype and therefore, the effects we observe are averaged across those genotypes. Upon removal from their population cages, flies were reared for two generations at a controlled density before use in experiments, to control for possible parental effects. Eggs for age-synchronised flies were collected over 18 h, and the resulting adult flies emerged during a 12 h window. They were then allowed to mate for 48 h before being anaesthetised with CO₂, at which point females were separated from males and allocated into experimental vials. Stocks were maintained and experiments were conducted at 25 °C on a 12 h: 12 h light:dark cycle at 65% humidity^30^.

For all lifespan assays, flies were placed into vials (FS32, Pathtech) containing 3 ml of treatment food at a density of ten flies per vial, with ten replicate vials per treatment. Flies were transferred to fresh vials every two to three days at which point deaths and censors were recorded and saved using the software package Dlife^31,32^. All experimental diets were made using a holidic medium, employing a constant nutritional background^20^ (also referred to as 100 N FLYAA^33^). Cholesterol was added to the diet at a concentration of either 0 g/l, 0.075 g/l or 0.3 g/l, following published protocols^16,19^, with 0.3 g/l cholesterol being considered the standard cholesterol concentration because it is not limiting for fecundity or lifespan.

### Intestinal barrier function analyses

A previously published protocol^21^ was used to assess intestinal barrier function. On day 12 from adult emergence, flies from each dietary treatment group were switched to experimental diets of the same recipe but with the addition of blue food dye (FD&C blue #1), at a concentration of 2.5% (wt/vol). Flies were then checked every second day for observable “smurfing”. This phenotype is caused by the blue dye escaping the gut lumen and entering the body cavity, which turns the whole fly blue. Flies were considered smurfs if their whole body turned completely blue (light smurfs and uncertain smurfs were excluded from the analyses)^21^. The blue dye did not modify the lifespan of flies (Fig. 1a, Supplementary Table 2). Once flies were identified as smurfing, they were separated and monitored until death occurred. Only living flies were classified as Smurfs.

### Immunohistochemistry

On day 18 (when flies fed low cholesterol diets had begun to die) guts were dissected from live flies (at least 3 per treatment) in ice-cold PBS and immediately fixed in 4% paraformaldehyde (Sigma) for 60 min at room temperature. After fixing, guts were washed with 0.1% Tween (PBST) then blocked for 30 min at room temperature in 2% heat-inactivated normal donkey serum in PBT. Guts were stained with DAPI at a concentration of 5 uL/mL and Phalloidin at a concentration of 14 uL/mL. All guts were mounted in Fluoromount-G (SouthernBiotech) and poly-L-lysine coated coverslips were used. Regions of interest in the midgut were then imaged using a confocal microscope (Olympus CV1000) with a 40x objective lens.

A minimum of 3 guts for each region of the gut, per condition were used. We examined the effects of cholesterol limitation on both the anterior (R1 and R2) and posterior regions (R4 and R5) of the midgut^23^. Guts were excluded from analyses if specimen or image quality were poor. An area of 150 µm × 150 µm (centred) was selected using Fiji, and then cells in this region were binned into scaled categories, where 1 = undisrupted honeycomb cells arranged with well aligned nuclei, 2 = a mixture of honeycomb structured cells and early-stage disorganisation, <50% of cells are elongated and irregular shaped, 3 = highly disorganised with >50% of cells are elongated and irregular shaped, and 4 = severe disruptions in epithelial cell organisation, all cells are misaligned, elongated and irregular in shape. These categories are adapted from a scoring system for epithelial disorganisation previously published^5^. These regions were selected as they are known to be impacted by dietary restriction^34^. All images were de-identified before scoring.

### Antibiotic assay

To examine the role of sepsis in death following loss of gut barrier function, antibiotics were added to food. Mated females were placed on a holidic medium (using the same P:C ratio and methods listed above) varying in cholesterol concentrations (0 g/l and 0.3 g/l) and antibiotic presence (Tetracylin, Ampicillin, Kanamycin, Erythromycin) for the duration of their lifespans. The antibiotic mix was delivered as described previously^26^ and contained kanamycin (50 μg/mL), ampicillin (50 μg/mL), tetracycline (10 μg/mL) and erythromycin (5 μg/mL). Stock solutions of each antibiotic were made in either MilliQ water (ampicillin and kanamycin) or ethanol (erythromycin and tetracycline) based on their solubility, such that adding 1 mL of stock to 1 L holidic media resulted in the above final concentrations. Immediately before dispensing the media, when it had cooled to ~60 °C, each antibiotic solution was added with a micropipette, under constant stirring. A separate length of tubing was used to dispense the food containing antibiotics, ensuring no antibiotics were inadvertently introduced to the antibiotic free media.

### Statistical analyses

All statistical analyses were performed using R Studio (Version 1.2.5042), available from http://www.R-project. org/). All data were analysed using Generalised Linear Mixed Effects Models (lme4^35^), Posthoc tests using the emmeans package^36^ were then applied to models for epithelial cell and smurf data.

## Supplementary information

Supplementary information

## Data Availability

All data is available at DOI:10.26180/28470362.
