# Supplementary material for: Short lifespan under dietary cholesterol depletion is associated with gut dysfunction in Drosophila melanogaster females
Source: NPJ Aging. 2026 Feb 19;12(1):46. doi: 10.1038/s41514-026-00341-5 (PMC13031265; doi:10.1038/s41514-026-00341-5)
Supplement: Supplementary file 1 — Supplementary information [file 41514_2026_341_MOESM1_ESM.pdf]

Supplementary Table 1. *A generalised linear model with replicate vial used as a random effect, reveals that cholesterol has a significant effect on smurfing.*

Smurfing	Chisq	DF	Pr(>Chisq)
Cholesterol	21.976	1	<0.001***

Supplementary Table 2. *The effects of cholesterol and blue dye on median lifespan were analysed using a linear mixed effects model, with replicate vial used as a random effect. There was a significant effect of cholesterol on lifespan, but no significant effect of blue dye, nor an interactive effect between cholesterol and blue dye.*

Median lifespan	Chisq	Df	Ps(>Chisq)
Cholesterol	345.99	2	<0.001***
Blue dye	3.65	1	0.060
Chol : Blue dye	3.45	2	0.177

Supplementary Table 3. The effects of cholesterol on smurfing were analysed using a Posthoc estimated marginal means (emmean) test on the linear-mixed effects model above (Table 1). Smurfing increased as cholesterol decreased.

Cholesterol (g/l)	emmean	SE	Df	lower.CL	Upper.CL	Group
0.3	0.163	0.097	20.5	-0.090	0.416	1
0.075	0.458	0.061	20.1	0.297	0.618	2
0	0.752	0.064	19.7	0.585	0.920	3

Supplementary Table 4. The effects of cholesterol on epithelial cell organisation in the midgut were analysed using a linear mixed-effects model, where gut id was used as a random effect. There was a significant effect of both dietary cholesterol and the interactive effect between cholesterol and gut region on epithelial cell disturbance

Midgut epithelial cell disturbances	Chisq	Df	Pr(>Chisq)
Cholesterol	10.732	1	<0.001***
Region	0.697	1	0.404
Cholesterol : Region	4.765	1	0.030*

Supplementary Table *5. The effects of cholesterol in the anterior and posterior regions of the midgut were analysed using a Posthoc estimated marginal means (emmean) test on the linear-mixed effects model above (Table 4). The effect of cholesterol on epithelial cell disturbance was significantly different to all other conditions in the anterior guts of flies fed 0.3g/l cholesterol.*

Region	Cholesterol (g/l)	emmean	SE	Df	lower.CL	Upper.CL	Group
Anterior	0.3	2.20	0.318	18.0	1.53	2.86	1
Posterior	0.3	3.42	0.282	20.5	2.83	4.01	2
Anterior	0.075	3.72	0.397	19.9	2.89	4.55	2
Posterior	0.075	3.66	0.252	17.4	3.13	4.19	2

Supplementary Table 6. *The effects of antibiotic administration on median lifespan were analysed using a linear mixed-effects model, with replicate vial used as a random effect. There was a significant effect of both antibiotic administration and cholesterol on lifespan, but no significant interactive effect between antibiotics and cholesterol.*

Median lifespan	Chisq	Df	Pr(>Chisq)
Antibiotics	8.907	1	0.003**
Cholesterol	190.511	1	<0.001***
Cholesterol : Antibiotics	0.026	1	0.872
